# Genome of *Herbaspirillum seropedicae* Strain SmR1, a Specialized Diazotrophic Endophyte of Tropical Grasses

**DOI:** 10.1371/journal.pgen.1002064

**Published:** 2011-05-12

**Authors:** Fábio O. Pedrosa, Rose Adele Monteiro, Roseli Wassem, Leonardo M. Cruz, Ricardo A. Ayub, Nelson B. Colauto, Maria Aparecida Fernandez, Maria Helena P. Fungaro, Edmundo C. Grisard, Mariangela Hungria, Humberto M. F. Madeira, Rubens O. Nodari, Clarice A. Osaku, Maria Luiza Petzl-Erler, Hernán Terenzi, Luiz G. E. Vieira, Maria Berenice R. Steffens, Vinicius A. Weiss, Luiz F. P. Pereira, Marina I. M. Almeida, Lysangela R. Alves, Anelis Marin, Luiza Maria Araujo, Eduardo Balsanelli, Valter A. Baura, Leda S. Chubatsu, Helisson Faoro, Augusto Favetti, Geraldo Friedermann, Chirlei Glienke, Susan Karp, Vanessa Kava-Cordeiro, Roberto T. Raittz, Humberto J. O. Ramos, Enilze Maria S. F. Ribeiro, Liu Un Rigo, Saul N. Rocha, Stefan Schwab, Anilda G. Silva, Eliel M. Souza, Michelle Z. Tadra-Sfeir, Rodrigo A. Torres, Audrei N. G. Dabul, Maria Albertina M. Soares, Luciano S. Gasques, Ciela C. T. Gimenes, Juliana S. Valle, Ricardo R. Ciferri, Luiz C. Correa, Norma K. Murace, João A. Pamphile, Eliana Valéria Patussi, Alberto J. Prioli, Sonia Maria A. Prioli, Carmem Lúcia M. S. C. Rocha, Olívia Márcia N. Arantes, Márcia Cristina Furlaneto, Leandro P. Godoy, Carlos E. C. Oliveira, Daniele Satori, Laurival A. Vilas-Boas, Maria Angélica E. Watanabe, Bibiana Paula Dambros, Miguel P. Guerra, Sandra Marisa Mathioni, Karine Louise Santos, Mario Steindel, Javier Vernal, Fernando G. Barcellos, Rubens J. Campo, Ligia Maria O. Chueire, Marisa Fabiana Nicolás, Lilian Pereira-Ferrari, José L. da Conceição Silva, Nereida M. R. Gioppo, Vladimir P. Margarido, Maria Amélia Menck-Soares, Fabiana Gisele S. Pinto, Rita de Cássia G. Simão, Elizabete K. Takahashi, Marshall G. Yates, Emanuel M. Souza

**Affiliations:** 1 Universidade Federal do Paraná, Curitiba, Brazil; 2 Universidade Estadual de Ponta Grossa, Ponta Grossa, Brazil; 3 Universidade Paranaense, Umuarama, Brazil; 4 Universidade Estadual de Maringá, Maringá, Brazil; 5 Universidade Estadual de Londrina, Londrina, Brazil; 6 Universidade Federal de Santa Catarina, Florianópolis, Brazil; 7 Embrapa Soja, Londrina, Brazil; 8 Pontifícia Universidade Católica do Paraná, Curitiba, Brazil; 9 Universidade Estadual do Oeste do Paraná, Cascavel, Brazil; 10 Instituto Agronômico do Paraná, Londrina, Brazil; Progentech, United States of America

## Abstract

The molecular mechanisms of plant recognition, colonization, and nutrient exchange between diazotrophic endophytes and plants are scarcely known. *Herbaspirillum seropedicae* is an endophytic bacterium capable of colonizing intercellular spaces of grasses such as rice and sugar cane. The genome of *H. seropedicae* strain SmR1 was sequenced and annotated by The Paraná State Genome Programme—GENOPAR. The genome is composed of a circular chromosome of 5,513,887 bp and contains a total of 4,804 genes. The genome sequence revealed that *H. seropedicae* is a highly versatile microorganism with capacity to metabolize a wide range of carbon and nitrogen sources and with possession of four distinct terminal oxidases. The genome contains a multitude of protein secretion systems, including type I, type II, type III, type V, and type VI secretion systems, and type IV *pili*, suggesting a high potential to interact with host plants. *H. seropedicae* is able to synthesize indole acetic acid as reflected by the four IAA biosynthetic pathways present. A gene coding for ACC deaminase, which may be involved in modulating the associated plant ethylene-signaling pathway, is also present. Genes for hemagglutinins/hemolysins/adhesins were found and may play a role in plant cell surface adhesion. These features may endow *H. seropedicae* with the ability to establish an endophytic life-style in a large number of plant species.

## Author Summary

In this work we describe the genome of *H. seropedicae* SmR1, a bacterium capable of fixing nitrogen and promoting the growth of important plant crops such as maize, rice, and sugar cane. Several investigations have shown that *H. seropedicae* supplies fixed nitrogen to the associated plant and increases grain productivity up to 50%. In the genome of *H. seropedicae*, we identified all the genes involved in the nitrogen fixation process and its regulation and, in addition, genes potentially involved in the establishment of efficient interaction with the host plant. Our analyses also revealed that this bacterium has a highly versatile metabolism capable of synthesizing and degrading a large number of organic and inorganic compounds. We believe that the knowledge of the genome of this bacterium will direct research to a better understanding of this important endophytic organism and allow the construction of new strains with enhanced agronomic efficiency.

## Introduction

Soil bacteria can interact in many ways with plant partners ranging from beneficial to pathogenic. Among beneficial interactions the rhizobia play a central role, forming symbioses with legume species to produce nitrogen-fixing nodules, which supply most of the required fixed nitrogen to many agriculturally important crops such as soybean, pea, beans and clover.

A now well-characterized class of diazotrophic bacteria capable of establishing endophytic associations and promoting plant-growth of important cereal and forage grasses such as wheat, rice and maize has been investigated in recent years. Among such well-known species are *Azospirillum brasilense*, *Gluconacetobacter diazotrophicus* and *H. seropedicae* [1]. The colonization of plant tissues by these bacteria may involve the interplay of many as yet unidentified biochemical signals and gene products from both partners. *H. seropedicae* is an aerobic, prototrophic, endophytic nitrogen-fixing, plant-growth promoting bacterium, of the *Betaproteobacteria* found inside tissues of important crops such as corn, sugar-cane, rice, wheat and sorghum without causing disease to the plant partner [2]–[9], and has a low survival rate in plant-free soil [5]. It fixes nitrogen under conditions of ammonium and oxygen limitation [5] and can express *nif* genes *in planta* [6]–[11]. Moreover, *H. seropedicae* is an active plant colonizer and has been shown to promote plant growth and increase grain production [4], [9], [12]. Aluminum tolerant varieties of rice were shown by the ^15^N_2_ dilution technique to incorporate significant amount of nitrogen derived from nitrogen fixation [4], [9]. Ecological, agronomic, physiological, genetic and biochemical aspects of this organism have been reviewed [1], [12]–[14].

## Results/Discussion

### General features

The genome of *H. seropedicae* strain SmR1, a spontaneous streptomycin resistant mutant of strain Z78 [15] (ATCC 35893) was sequenced and annotated by the Paraná State Genome Programme (Genopar Consortium, www.genopar.org). Reads from the Sanger automatic sequencing (125,000) and from a full 454 FLX Titanium Roche Pyrosequencer run (1,220,352), corresponding to 100 times the coverage of the estimated genome size, were assembled to produce the genome sequence. End-sequencing of approximately 700 cosmids with an average insert of 40 kb was used to validate the final assembly.

**Figure 1:**
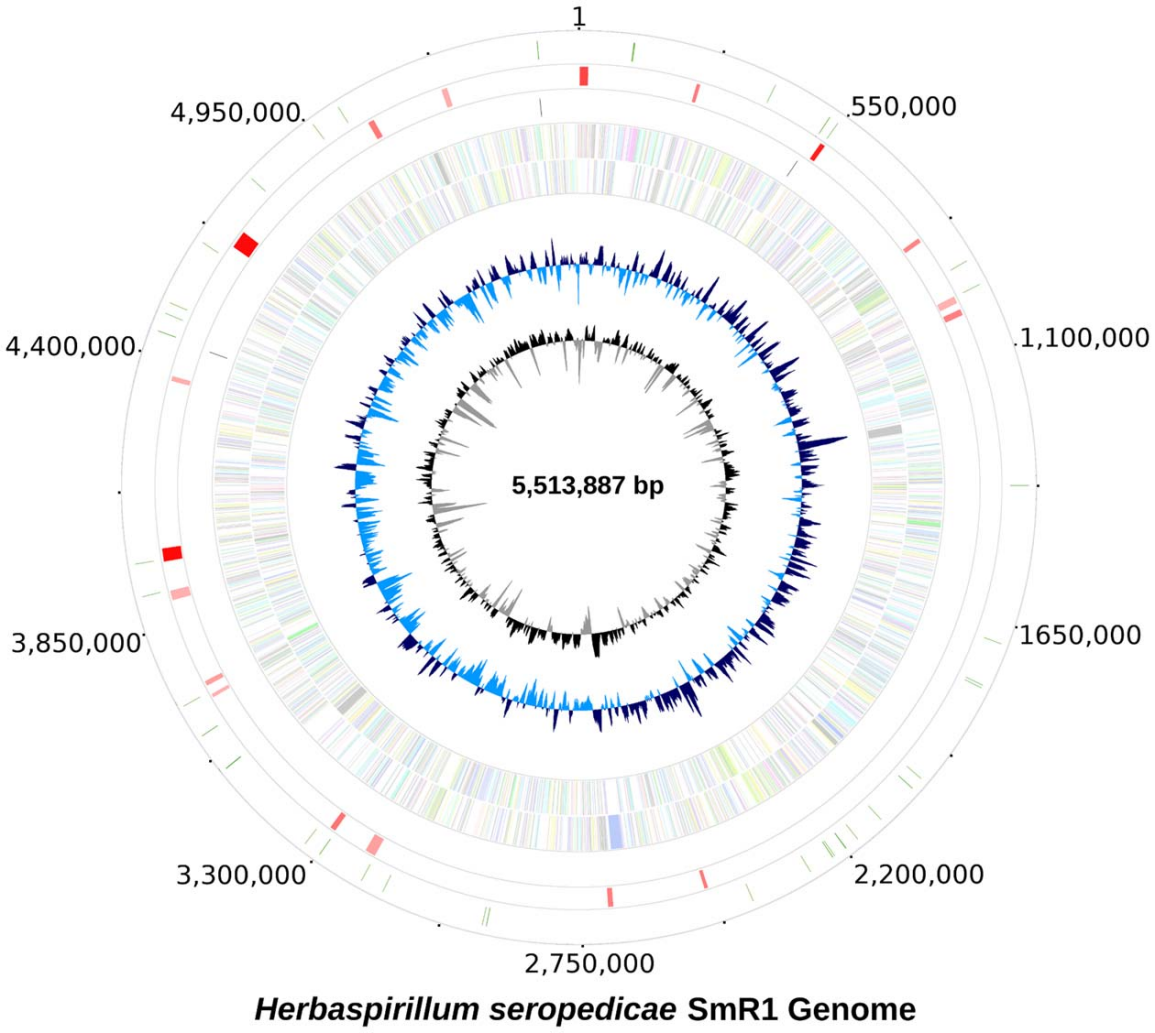
The genome of *Herbaspirillum seropedicae* SmR1. From inside to outside 1) G+C content; 2) GC skew; 3) genes color-coded according the COG functional categories; genes in the + strand and − strand are represented in the inside and outside circles respectively; 4) rRNAS operons; 5) putative horizontally transferred regions identified using IVOM: light red indicates low score and dark red indicates high score; 6) regions of *H. seropedicae* genome identical to castor bean (*Ricinus communis*) sequences (minimum of 200 bp in length and higher than 90% in identity).

The genome consists of a single circular chromosome of 5,513,887 base pairs with 63.4% G+C content (Table 1) and a total of 4,735 potential ORFs, encoding 3,108 proteins with assigned functions, 497 with general function prediction only and 1,130 with no known function, covering 88.3% of the genome. Coding sequences for 55 tRNA representing all 20 protein amino acids were also identified. The genome has 3 complete *rRNA* operons, one in the positive and two in the negative strand, all containing a pair of Ile-tRNA/Ala-tRNA genes in the intergenic region between the 16SrRNA and 23SrRNA genes (Figure 1). Genes for 19 of the 20 aminoacyl-tRNA synthetases are present with the exception of a gene coding for asparaginyl-tRNA synthetase. The biosynthesis of aspartyl-tRNAAsn or glutamyl-tRNAGln occurs via transamidation catalysed by an Asp-tRNAAsn/Glu-tRNAGln amidotransferase, an enzyme coded by the *gatBAC* operon as in most Bacteria [16]. These genes are widely spread among bacteria and are found in the genomes of other closely-related *Betaproteobacteria* such as *Herminiimonas arsenicoxydans*, bacteria of the *Burkholderia* genus, and *Minibacterium massiliensis (Janthinobacterium species Marseille)*.

**Table 1:** General features of the genome of *Herbaspirillum seropedicae* SmR1.

Size, bp	5,513,887
G+C %	63.4
Total number of genes	4804
Total number of CDS	4735
Average ORF size, bp	1028.8
Protein coding regions (%)	88.3
rRNA operons	3
tRNAs	55
Genes with functional assignment	3108
General function prediction	497
Function unknown	1130
Putative horizontally-transferred regions	18

The probable origin of replication was identified based on the GC skew [17] and the positions of the genes *dnaA*, *dnaN* and *gyrB*. It maybe contained in the *dnaA*-*dnaN* intergenic region or upstream *dnaA*, where DnaA binding sequences were found. The region upstream of *dnaA* is unique, since instead of the *rpmHrnpA* operon present in most *Proteobacteria* it contains a probable glutamine amido transferase type II gene. Downstream from the *dnaA*, *dnaN* and *gyrB* genes there is a low G+C content (52%) region spanning 16.6 kbp of probable lateral transfer origin containing a reverse transcriptase gene of bacterial retrotransposons (RT_Bac_retron_I).

In the *H. seropedicae* genome 18 regions of probable lateral transfer origin, such as insertion sequences and phages were found. The two largest regions contain genes of bacteriophage origin. Region 1 (213,067 to 238,374) has a higher G+C content (66.4%) than the genome and contains 33 ORFs related to phage capsid assembly, regulation and phage transcription. Region 2 (967,869 to 1,006,417) has a lower G+C content (58.1%) with 52 ORFs, many related to phage P2.

One of the low G+C content (53.9%) regions contains a plasmid addiction module (operon *phd/doc*) coding for the PHD (prevents-host-death) and DOC (death-on-curing) proteins constituting a toxin-antitoxin (TA) module [18]. There are 3,412 PHD (http://www.ebi.ac.uk/interpro/IEntry?ac=IPR006442) and 707 DOC (http://www.ebi.ac.uk/interpro/IEntry?ac=IPR006440) protein sequences described in the Domain Bacteria, suggesting the widespread occurrence of this protection mechanism. ‘Toxin–antitoxin (TA) modules’ have been recognized as playing important roles in bacterial stress physiology and genome stabilization [18]–[20].

Genes coding for two partial and two complete transposases and 5 phage-related (three complete and two partial) and two genomic (*xerC* and *xerD*) recombinases/integrases were found in the *H. seropedicae* genome. The relative few number of genes related to mobile elements seems to be a common feature in all the genomes of endophytic bacteria (http://www.expasy.ch/sprot/hamap/interactions.html#Plant_endophyte?) sequenced to date. The exception is *G. diazotrophicus* Pal5, with 138 transposases and 223 insertion sequences [21]. The paucity in the number of putative transposable elements may suggest a low recombination/rearrangement in the genome of *H. seropedicae* SmR1, possibly reflecting its evolutionary adaptation to an endophytic lifestyle, and indicating a low rate of recent gene transfer that is presumably due to adaptation to a stable microenvironment, as suggested by Krause et al. [22] for the genome of *Azoarcus* sp.strain BH72.

### General metabolism

*H. seropedicae strain* SmR1 is capable of growing on mono-saccharides such as D-glucose, D-fructose, D-galactose and L-arabinose, with sugar alcohols and organic acids such as L-malate or L-lactate but failed to grow on oligo- or polysaccharides [5], [15]. Accordingly, the genome of *H. seropedicae* contains the complete set of genes for the Entner-Doudoroff and pentose phosphate pathways. The Embden-Meyerhoff-Parnas (EMP) pathway lacks the gene coding for the classical 6-phosphofructokinase (PFK, E.C. 2.7.1.11), suggesting that *H. seropedicae* probably requires the involvement of the Entner-Doudoroff and the pentose phosphate pathways to metabolize D-glucose, D-fructose or D-mannose to pyruvate via the EMP pathway. Several ABC-type sugar-transport systems and one PEP/PTS transport system are present in the genome of *H. seropedicae*, consistent with its capacity to grow on a large number of monossaccharides [5], [15]. *H. seropedicae* has all the genes needed for gluconeogenesis: the EMP pathway plus those coding for fructose-1,6-biphosphatase, phosphoenolpyruvate dikinase, D-lactate and L-lactate dehydrogenases, and from two-carbon substrates such as ethanol via the glyoxalate cycle.

All genes necessary for the metabolism of D-galactose via 2-dehydro-3-deoxy-D-galactonate-6-phosphate leading to pyruvate and D-glyceraldehyde-3-phosphate are present in the genome. Subsequent conversion of pyruvate to acetyl-CoA is via the pyruvate dehydrogenase complex, while lactate dehydrogenase serves as an entry point of lactate during lactate-dependent growth. The conversion of 2-dehydro-3-deoxy-L-arabinonate to 2-keto-glutarate involves the sequential action of a dehydrase and NAD(P)-dehydrogenase. No such specific enzymes were found although several dehydrases and dehydrogenases are present in the genome of *H. seropedicae* SmR1.

The pathway for L-arabinose metabolism was shown to involve non-phosphorylated intermediates to produce 2-ketoglutarate [23]. This pathway probably involves the enzymes of the D-galactose breakdown pathway due to the identical configuration of C-2, C-3 and C-4 to those of L-arabinose.

The genome of *H. seropedicae* has all the genes for the citric acid cycle. Pathways replenishing intermediates of the cycle include the glyoxylate cycle (isocitrate lyase and malate synthase), the complete fatty acid β-oxidation pathway, the malic enzyme, phosphoenolpyruvate carboxykinase, phosphoenolpyruvate carboxylase and, from the degradation of the L-aminoacids alanine, glutamate, aspartate, asparagine and glutamine.

*H. seropedicae* grows in ethanol-containing media via alcohol dehydrogenase and aldehyde dehydrogenase to yield acetyl-CoA which can feed into the citric acid cycle.

*H. seropedicae* is an aerobic bacterium capable of fixing nitrogen under conditions of oxygen limitation. The genome of *H. seropedicae* has genes for four terminal oxidases: cytochrome c oxidase aa3 and the three alternative terminal oxidases bd, cbb3 and o, suggesting a branched respiratory chain. It has all the genes for the synthesis of NADH dehydrogenase, succinate dehydrogenase, cytochrome c reductase and also the complete set of genes for ATP synthase. The high affinity terminal oxidase cbb3 presumably supports ATP synthesis under the limiting oxygen conditions essential for nitrogenase synthesis and activity, as in other aerobic diazotrophs [24].

### Polybetaalkanoates

*H. seropedicae* SmR1 synthesizes poly(3-hydroxybutyrate) under diazotrophic growth conditions and, as in other bacteria, it can reach up to 60% of the cell dry weight [25]. *In silico* analysis of the genome of *H. seropedicae* revealed 13 genes potentially involved in poly(3-hydroxybutyrate/alkanoate) synthesis and degradation. A main cluster containing *phbF*, *phbB* and *phbC* coding respectively for a transcription regulator, acetoacetyl-CoA reductase and poly(3-hydroxybutyrate) synthase was found between bases 3,411,979 and 3,415,628. In addition there are three *phbA* (acetyl-CoA acyltransferase), one *phbC* (poly(3-hydroxybutyrate) synthase), two *phaC* (poly(3-hydroxyalkanoate) synthase), one *phaB* (3-keto-acyl-CoA reductase), two *phaP* (phasin) and two poly3-hydroxyalkanoate depolymerase (*phaZ*) genes. The data suggests the presence of two systems for the synthesis of poly(3-hydroxyalkanoate) and one specific for poly(3-hydroxybutyrate) in *H. seropedicae* strain SmR1, which is consistent with the isolation of poly(3-hydroxybutyrate) and poly(3-hydroxybutyrate/valerate) co-polymer from strain Z67 [25].

### Amino acid metabolism

The genome of *H. seropedicae* contains genes coding for the synthesis of all 20 protein amino acids. However, it has limited ability to grow on amino acids as carbon sources. It can grow on L-proline, L-tyrosine, D/L-alanine, β-alanine, L-isoleucine and L-glutamate but failed to grow on L-phenylalanine, L-histidine, L-arginine or L-lysine [5], [15], [26], [27]. *In silico* analysis of the genome content suggested that the pathway for the degradation of L-histidine and L-lysine is incomplete. No specific L-arginine transporter was found, supporting the observation that this molecule cannot serve a sole N-source for *H. seropedicae* growth [26]. On the other hand, endogenously synthesized L-arginine can be catabolised to agmatine, putrescine and to 4-aminobutanoate which could be further converted to succinate in *H. seropedicae*. A strain of *H. seropedicae* carrying a Tn5-*lacZ* insertion in the *speB* gene coding for arginase is induced under low ammonium conditions [28], suggesting the presence of a second pathway for arginine degradation under conditions of ammonium limitation.

### Urea metabolism

*H. seropedicae* is capable of synthesizing and degrading urea. Genes coding for the complete urea cycle enzymes, the probable pathway for arginine biosynthesis in this bacterium, using proline and carbamoyl phosphate as a precursors were found. Urea is degraded by urease. The urease operon contains the structural genes *ureA*, *ureB* and *ureC* and the accessory genes *ureD*, *ureE*, *ureF*, *ureG* and *ureJ*. This operon is very similar to that of *Janthinobacterium* sp. Marseille, although it is lacking in the *Herminiimonas arsenicoxidans* genome. A complete ABC-type urea transport operon (*urtABCDE*) was found upstream from the *ure* gene cluster in both the *H. seropedicae* and *Janthinobacterium* genomes, similar to that of *Corynebacterium glutamicum* [29]. Analysis of a mutant strain of *H. seropedicae* Z78, containing a Tn5-*lacZ* insertion in the *urtE* gene and obtained by random Tn5-*lacZ* insertion and screening for differential expression under N-limiting conditions, led to the suggestion that both the *urt* and *ure* genes are expressed under N deprivation [28] and are probably controlled by the Ntr system since a σ^54^-dependent promoter is located upstream of *urtA*.

### Nitrogen fixation

The nitrogen fixation genes (*nif*) of *H. seropedicae*, including *nifA, nifB, nifZ, nifZ1, nifH, nifD, nifK, nifE, nifN, nifX, nifQ, nifW, nifV, nifU and nifS* were found in a region spanning 37,547 bp interspersed with *fix, mod, hes, fdx, hsc and other genes*. The 46 ORFs of this cluster are organized in 7 NifA-, σ^54^-dependent operons. This cluster is flanked by two 348 bp fragments 93% identical (325 out of 348 bp), probably derived from a partial duplication of the *gloA* gene, corresponding to the region coding for the 99 aminoacid residues of the C-terminus of GloA. Just upstream the *nif* cluster a sequence reminiscent of a transposase gene is present, as in the *nif* cluster of *Burkholderia vietnamiensis* strain G4 chromosome 3. These are suggestive that *H. seropedicae* acquired the *nif* cluster by lateral transfer.

Two globin-like genes expressed from a putative NifA-regulated promoter are present in the *nif* cluster of *H. seropedicae*, while a single globin-like gene is found in this cluster in *Burkholderia xenovorans* LB400 chromosome 2 and *Burkholderia vietnamiensis* G4 chromosome 3. Since NifA in *H. seropedicae* is transcriptionally active only under limiting oxygen tensions, these globin-like proteins may support the delivery of oxygen to energy production under nitrogen-fixing conditions in both the free-living and the endophytic state. The *nif* cluster carries all the genes necessary for nitrogenase synthesis and activity, including molybdenum uptake, electron transport and metal cluster synthesis, and the *nif* operon regulatory gene, therefore a cluster capable of endowing an organism with the full capacity to fix nitrogen. No genes for alternative nitrogenases nor for both hydrogenase types were found in the genome of *H. seropedicae*.

### Nitrate metabolism

*H. seropedicae* is capable of growing aerobically with nitrate as sole N source, but is unable to denitrify anaerobically [15]. *In silico* analysis revealed that the genome of *H. seropedicae* contains the genes for an assimilatory and a dissimilatory nitrate reductase. The genes for nitrate assimilation are located in two genomic regions: the first contains the genes for the ABC-type nitrate transport (*nasFED*) and the second contains the gene *narK*, a nitrate/nitrite transporter, *nirBD* coding for the assimilatory nitrite reductase and *nasA* the structural gene for the assimilatory nitrate reductase. In the same operon, upstream of *nasA*, a gene coding for a probable FAD-dependent pyridine nucleotide-disulphide oxidoreductase could fulfill the function of NasC in *H. seropedicae*. This latter operon organization is common in the *Ralstonia eutropha* H16, *R. solanacearum* and *R. metallidurans* genomes. The complete set of *narGHJI* genes coding for a respiratory nitrate reductase is present in *H. seropedicae* located downstream from two nitrate/nitrite transporters *narK1* and *narU* and upstream of the regulatory pair *narXnarL*. No genes coding for dissimilatory nitrite reductase, nitric oxide reductase or nitrous oxide reductase are present in *H. seropedicae* SmR1. This is consistent with the observation by Baldani et al. [15] who found no evidence of denitrification as the release of N_2_O by *H. seropedicae* compared with that from *Azospirillum lipoferum*.

Presumably the nitrite formed by this respiratory nitrate reductase can only be converted to ammonium ions and not dissimilated to N_2_. The role of this dissimilatory nitrate reductase in *H. seropedicae* is not clear, however, it may be involved in NO production and in survival under hypoxia as described for *Mycobacterium tuberculosis* [30]. A gene coding for a nitric oxide dioxygenase was found in the genome of *H. seropedicae* transcribed in the opposite direction to the *norR* gene located immediately upstream. Nitric oxide mediates plant defense responses against pathogens and is used as a signaling molecule [31]–[34] and the role of this nitric oxide dioxygenase may be NO detoxification during the initial stages of the *H. seropedicae* endophytic colonization of plants.

### Plant–bacterial interaction

*H. seropedicae* is capable of the rapid colonization of several Gramineae [11]. Monteiro et al. [35] showed *H. seropedicae* in cortical cell layers of maize roots 12 hours after inoculation and xylem occupation after 24 hours. Three important aspects of the *H. seropedicae* beneficial association with plants are its ability to invade and colonize plant hosts, to thrive on plant exudates and to benefit associated plants. Interestingly, genes coding for plant cell wall degradation enzymes such as glycosidases, cellulases and hemi-cellulases associated with bacterial penetration were not found in the *H. seropedicae* genome. It is likely therefore that this organism relies only on natural discontinuities of the plant root epidermis for penetration as suggested by Olivares et al. [6].

During the annotation of the *H. seropedicae* genome a large number of Blast returned hits with high levels of identity to ESTs and genomic sequences of *Ricinus communis*. A total of 686 of such sequences was found: these varied in size from 100 to 2000 bp and were distributed randomly on the genome. This result suggests that the ricinus plant used to construct the libraries had an active *Herbaspirillum* endophyte. Recently we showed that *H. seropedicae* can colonize *Phaseolus vulgaris* [36], and *Herbaspirillum lusitanum* was isolated from *Phaseolus* nodules [37]. Together these results indicate that *Herbaspirillum* species may have a broader host range than previously described.

### Protein secretion

The genome of *H. seropedicae* has genes involved in Sec-dependent and Sec-independent protein export systems. The Sec-dependent secretion systems present are type II (T2SS), type V (auto-transporters; T5SS) and the type IV *pili*, while the Sec-independent secretion systems are type I (ABC transporters), type III (T3SS) and the type VI (T6SS). T3SS, type IV *pili* and T6SS have been implicated in delivering toxic effector proteins directly into the cytoplasm of eukaryotic cells by pathogenic bacteria. In non-pathogenic bacteria, such as *H. seropedicae*, the latter secretion systems may be involved in plant–bacterial recognition. In addition to the Sec translocase system, twin arginine translocase (*tat* genes) are also present in the *H. seropedicae* SmR1 genome. Genes for the type IV secretion system are absent from the *H. seropedicae* genome.

**Figure 2:**
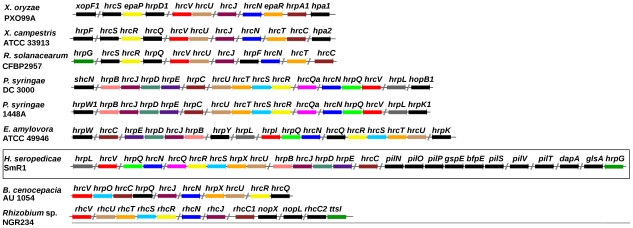
The type III secretion system gene cluster of *H. seropedicae* SmR1 and other organisms. Genes of the same color in different organisms are homologous. Genes colored in black have no counterpart in the genomic regions shown.

Effector proteins delivered by the T3SS of pathogenic bacteria can circumvent plant defense mechanisms and control host metabolism to their advantage. However, the T3SS system may also optimize beneficial host-bacteria interactions, a phenomenon first demonstrated for *Rhizobium* NGR234 which secrete effector proteins via the T3SS in response to flavonoids exudated by the plant host roots. The effect of the secreted effector can either enhance or diminish nodulation depending on the host legume [38]–[40]. In the *H. seropedicae* genome the T3SS gene region, potentially involved in plant/bacterial interactions, spans a 22 kb region of DNA which contains 7 *hrp* (hypersensitive response and pathogenicity), 8 *hrc* (hypersensitive response conserved), and 11 hypothetical ORFs (Figure 2). Two protein T3SS related genes *hrpG*, coding a transcription activator, and *hpaB*, that codes for a chaperone involved in protein secretion, are found at 10 kb downstream from the *hrp/hrc* cluster. The G+C content of the *hrp/hrc* region (66.1%) is slightly higher than the chromosomal average of 63.4%. Furthermore, no transposition elements flanking this region are present, suggesting that this region is not a recent acquisition by *H. seropedicae* or was laterally transferred from a closely related species. Gram negative bacteria that contain the *hrp* genes are divided into two main groups, according to the regulatory circuitry controlling T3SS gene expression and organization. In group I *hrp* genes are regulated by HrpL, a member of the ECF family of alternative sigma factors [41]–[43]. Induction of the *hrpL* gene requires the σ^54^ activator HrpS (*Erwinia* spp., *Pantoea stewartii*), or HrpS and HrpR (*P. syringae*). In organisms of group II the *hrp* genes are activated by an AraC-like activator, HrpB (*R. solanacearum*) or HrpX (*Xanthomonas* spp) [44]–[46], and the *hrpX* and *hrpB* genes are activated by the HrpG protein [46], [47]. *H. seropedicae* contains a gene for the ECF-like sigma factor HrpL resembling group I bacteria such as *Pseudomonas syringae*, *Erwinia amylovora*, and *Pantoea stewartii*. In contrast, *H. seropedicae* contains a gene for the HrpG protein, a transcriptional activator characteristic of group II bacteria, suggesting a hybrid regulatory system, involving regulatory elements from both groups. In addition, *hrp*-box motifs were found upstream of the *hrp/hrc* operons.

Contiguous to the *hrp/hrc* cluster were found the genes *pilNOPgspEbfpEpilSVTdapAglsA* (Figure 2). These code for proteins of the type IV *pili*, a system responsible for processes such as attachment to surfaces, twitching motility, biofilm formation, virulence and protein secretion [48]–[50]. In this region a gene coding for a lytic transglycosylase was also found. This protein is probably involved in partial degradation of the peptidoglycan to allow the efficient assembly and anchoring of supramolecular transport complexes such as T2SS, T3SS and type IV *pili* to the cell envelope. Interestingly, downstream from the genes of the type IV *pili* a methionyl-tRNA gene is present, suggesting that the *hrp/hrc*-type IV *pili* genes may form a genomic island.

A proteomic investigation of the secretome of *H. seropedicae* grown in minimal medium indicated a large number of proteins involved in cellular processes (45.4%), metabolism (36%), and hypothetical and conserved hypothetical (14.1%) proteins [51]. However, no type III proteins were detected among the secreted proteins, suggesting that specific physiological conditions may be required for expression and activity of the T3SS and synthesis of effector proteins in *H. seropedicae*.

### Osmotic stress

A probable operon involved in the synthesis and degradation of a homopolymer of D-glucose, composed by the genes *glgA* (glycogen synthase), *glgB* (1,4-alpha-glucan branching enzyme), *glgX* (glycogen debranching enzyme), *treZ* (malto-oligosyltrehalose trehalohydrolase), *malQ* (4-alpha-glucanotransferase) and *treY* (malto-oligosyl trehalose synthase), is located in the complementary strand spanning bases 2,843,031 to 2,856,518. Neighbor gene analysis using the String server (http://string-db.org) revealed a similar gene organization in the *Alphaproteobacteria Rhizobiales*, in the *Betaproteobacteria Burkholderia* spp. and *Gammaproteobacteria Xanthomonas* spp. Synthesis of an amylopectin-like polysaccharide may be related to osmotic stress protection and energy storage. This may reflect the potential of these bacteria to interact with plants in an endophytic or pathogenic mode. Environmental *Oxalobacteraceae* such as *Janthinobacterium* sp. (strain Marseille) (*Minibacterium massiliensis*) and *Heminiimonas arsenicoxidans*, of the same family as *H. seropedicae*, lack these genes.

There are genes for two trehalose synthesis systems in the genome of *H. seropedicae*. One of these involves *otsA* coding an alpha,alpha-trehalose-phosphate synthase (UDP-forming) and *otsB*, coding a trehalose-6-phosphate phosphatase, and a glucoamylase gene. The other system involves an alpha-amylase (Hsero_2325), trehalose synthase (Hsero_2326), and a 1,4-alpha-glucan branching enzyme (Hsero_2327) and constitute an operon.

Furthermore, four Na^+^(K^+^)/H^+^ antiporter (*nhaA*, *nhaP*, *arsB* and Hsero_3967) genes are present in the genome of *H. seropedicae* which may contribute to the defense against osmotic/saline stress.

### Polyphosphate

Polyphosphates are involved in the response of bacteria to extreme stress conditions of salinity, osmolarity, desiccation, N-starvation, UV radiation, barometric pressure, pH, and temperature [52]. Two genes coding for polyphosphate kinase (Hsero_0611 and *ppk*), the enzyme responsible for the synthesis of polyphosphate, and one coding for an exopolyphosphatase (*ppx*), are present in the genome of *H. seropedicae*. These systems may constitute adaptative defense mechanism for the endophytic life style of *H. seropedicae*.

### Siderophores

The rhizosphere and the rhizoplan are highly competitive areas for bacterial survival and development; the capacity to acquire siderophores complexed with Fe^3+^ in Fe-limited soils would be advantageous in such competition. *H. seropedicae* has at least 27 genes involved in iron transport and metabolism. A very large gene (27,483 bp) coding for a modular peptide synthase is the only protein of *H. seropedicae* probably involved in siderophore synthesis (Hsero_2343). This gene is located downstream from *cirA*, a TonB-dependent siderophore receptor, and *prfI*, an ECF sigma factor. The genome has 17 TonB-dependent siderophore receptors and one ABC-type hydroxamate-type ferric siderophore uptake system. Presumably iron uptake is via active transport involving an ABC-type system and TonB/ExbB/ExbD. The rice endophyte *Azoarcus* also contains a plethora of TonB dependent siderophore receptors [22], [53]. This large number of iron receptors may endow organisms such as *H. seropedicae* and *Azoarcus* with a high competitiveness in iron-limited environments and may confer the ability to out-compete other bacteria. Also present in the genome is the global iron regulator gene *fur*.

### Auxin biosynthesis

The plant growth-promoting bacteria probably owe some of their ability to the production and secretion of phytohormones [54]. There are four possible pathways in *H. seropedicae* for the production of indoleacetic acid (IAA) from tryptophan. The most probable route is via indolepyruvate, to indole-acetic acid catalysed by tryptophan transaminase and indolepyruvate ferredoxin oxidoreductase. Genes for the other possible metabolic routes are also present: 1) tryptophan to indoleacetate via indoleacetamide; 2) from indoleacetamide to indoleacetate via indoleacetonitrile and 3) tryptophan to indoleacetate via tryptamine and indoleacetaldehyde.

### Modulation of endogenous ethylene levels by ACC deaminase

Ethylene is a known plant hormone synthesized from S-adenosylmethionine by 1-aminocyclopropane 1-carboxylate (ACC) synthase, an enzyme activated by IAA under biotic and abiotic stress conditions [55]. ACC is converted to ethylene by ACC oxidase. A gene coding ACC deaminase is present in the *H. seropedicae* genome and is known to compete with ACC oxidase, modulating the levels of ethylene in plants, thus decreasing the stress response promoted by ethylene and allowing plant growth under stress conditions [56]. The coordinated production of IAA and ACC deaminase by *H. seropedicae* is a likely mechanism for plant growth promotion by this microorganism as shown for the *Herbaspirillum*-related endophytic, nitrogen-fixing, plant growth-promoting *Betaproteobacterium*, *Burkholderia phytofirmans* PsJN [57].

### Metabolism of aromatic compounds

**Figure 3:**
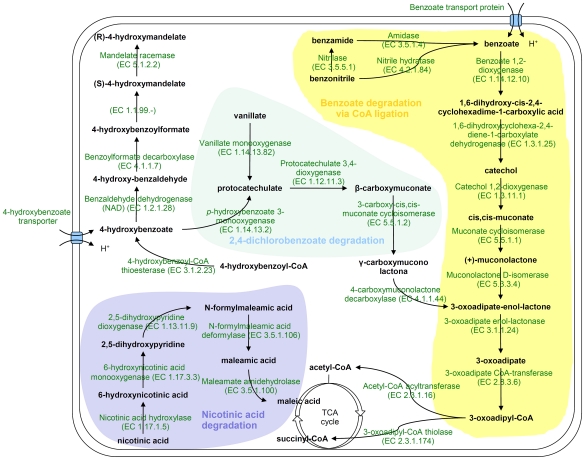
Proposed pathways for aromatic compounds metabolism in *H. seropedicae* SmR1.

*H. seropedicae* genome contains genes coding for degradation of benzoate, benzamide, benzonitrile, hydroxy-benzoate, and vanillate (Figure 3). In separate clusters, genes coding for a nicotinic acid degradation pathway similar to that of *Pseudomonas putida* [58] and a *meta* pathway of an as yet unknown phenolic compound were found. These pathways may be important to allow *H. seropedicae* to thrive on plant tissues by conferring both metabolic flexibility and defense against plant-derived toxic chemicals.

### Hemagglutins/hemolysins

Hemagglutins/hemolysins are cytotoxic proteins implicated in animal pathogenesis, but a large number of genes coding for such proteins have been found in plant pathogens and plant-interacting bacteria [59]. Twenty genes related to hemagglutinin/hemolysin are present in the genome of *H. seropedicae* SmR1, and 9 additional genes code for hemagglutinin/hemolysin accessory proteins such as transporters/activators. Three genes code for hemagglutinins with adhesin-like domains, two of which are homologous to *fhaB* of *Xanthomonas axonopodis* pv *citri* [59] and are associated with genes coding for the accessory FhaC protein. The products of these genes may be required for surface attachment and biofilm formation during plant tissue colonization [60].

### Concluding remarks

**Figure 4:**
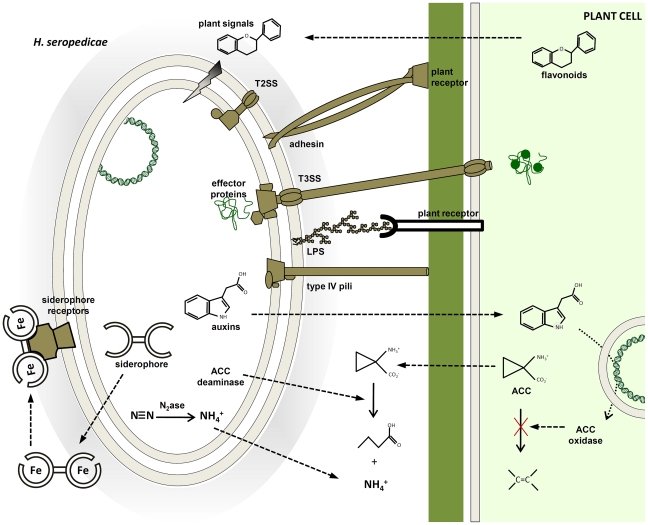
Molecular mechanisms probably involved in plant colonization and plant growth promotion identified in the *H. seropedicae* SmR1 genome. Plant signals can modulate the expression of bacterial genes coding for adhesins, type IV *pili* and enzymes of lipopolysaccharide (LPS) synthesis, triggering bacterial attachment to root surfaces. The molecular communication involves bacterial protein secretion and phytohormones to stimulate plant growth and modulate plant defense response. In addition, modulation of plant ethylene levels by ACC deaminase may contribute to plant growth promotion. The success of the endophytic association depends on a compatible genetic background that leads to benefits for both organisms.

The genome of *H. seropedicae* revealed a metabolically versatile bacterium, with the ability to thrive on a range of plant metabolites from sugars to phenolic compounds (Figure 4). It is capable of synthesizing plant-growth modulators such as auxins and gibberellins, although only potential pathways for IAA synthesis were found in the genome; the cryptic genes for gibberellins and citokinins syntheses remain to be identified. It is surprising that an aggressive plant colonizer such as *H. seropedicae* [35] is devoid of glycohydrolases involved in plant cell wall degradation. However, *H. seropedicae* displays an impressive variety of protein secretion systems and hemagglutinins/hemolysins/adhesins that may facilitate plant invasion, colonization and an endophytic life, following penetration through natural epidermal wounds. Additional contributors to the plant-growth-promoting capacity of *H. seropedicae* may depend on the many genes involved in nitrogen fixation, NO_3_^−^ and NO_2_^−^ assimilation, NO oxidation, and ACC deamination (Figure 4). The presence of ACC deaminase may modulate ethylene production stimulated by IAA from bacterial origin, thus allowing plant resistance to biotic and abiotic stress conditions. These non-specific plant-interaction systems may endow *H. seropedicae* with the ability to establish an endophytic life-style in a large number of plant species.

## Materials and Methods

### Organisms and DNA purification

*H. seropedicae* strain SmR1 was grown in liquid NFbHP medium containing 20 mM NH_4_Cl and 0.5% potassium malate, as described by Klassen et al. [26]. DNA was purified using phenol-chloroform extraction of cells lysed with lysozyme and SDS.

*E. coli* strain hosts XL1-Blue and DH10B were grown in LB or Terrific broth [61].

### Genome sequencing and assembly

The genome sequence of *H. seropedicae* strain SmR1 total DNA was determined by the whole genome sequencing strategy [62] using short fragment (1.5–3.0 kb) libraries in pUC18 and pUC19 (Amersham Biosciences) and cosmid libraries in Supercos (Promega). DNA inserts were sequenced using the DYEnamic ET kit (GE HealthCare) and MegaBace 1000 automatic sequencers. Plasmid and cosmid DNA template preparation was performed by alkaline lysis and sequenced in 96-well plates according to standard procedures. A full DNA sequence run was performed in a Roche 454 GS-FLX Titanium by Creative Genomics, USA.

The genome was assembled using the Phred/Phrap/Consed package (www.phrap.org) and the Roche NewBler assembler. End sequences of cosmids were used to validate the genome assembly. Contig scaffolding was suggested by Autofinisher (www.phrap.org) and gaps were closed using PCR and whole insert sequencing of selected plasmid clones. The average final Phred score value was higher than 70.

### Genome annotation

Potential protein coding regions (ORFs) were identified by an integrated automatic annotation platform with Glimmer 2 [63], [64], and Blast softwares [65]. Probable functions of translation products of potential orfs were inferred using the Blast package to search the public databases GenBank (L), COG [66], KEGG [67] and pFAM [68]. The output of an in-house annotation platform was reviewed by human annotators for gene assignment and proposed function. Each proposed gene sequence and annotation was validated with individual inspection by Artemis V11 [69]. tRNAs were located using tRNAscan-SE [70]. Ribosomal RNA operons were located using Blastn [65]. Putative horizontally transferred DNA regions were identified using IVOM [71].
